# Probiotics Surface-Delivering Fiber2 Protein of Fowl Adenovirus 4 Stimulate Protective Immunity Against Hepatitis-Hydropericardium Syndrome in Chickens

**DOI:** 10.3389/fimmu.2022.919100

**Published:** 2022-06-28

**Authors:** Zhipeng Jia, Xinghui Pan, Wenjing Zhi, Hang Chen, Bingrong Bai, Chunli Ma, Dexing Ma

**Affiliations:** ^1^ College of Veterinary Medicine, Northeast Agricultural University, Harbin, China; ^2^ College of Food Science, Northeast Agricultural University, Harbin, China; ^3^ Heilongjiang Key Laboratory for Experimental Animals and Comparative Medicine, Northeast Agricultural University, Harbin, China

**Keywords:** FAdV-4, Fiber2, Lactococcus lactis, Enterococcus faecalis, oral immunization

## Abstract

**Background and Objectives:**

Hepatitis-hydropericardium syndrome (HHS) caused by Fowl adenoviruses serotype 4 (FAdV-4) leads to severe economic losses to the poultry industry. Although various vaccines are available, vaccines that effectively stimulate intestinal mucosal immunity are still deficient. In the present study, novel probiotics that surface-deliver Fiber2 protein, the major virulence determiner and efficient immunogen for FAdV-4, were explored to prevent this fecal–oral-transmitted virus, and the induced protective immunity was evaluated after oral immunization.

**Methods:**

The probiotic *Enterococcus faecalis* strain MDXEF-1 and *Lactococcus lactis* NZ9000 were used as host strains to deliver surface-anchoring Fiber2 protein of FAdV-4. Then the constructed live recombinant bacteria were orally vaccinated thrice with chickens at intervals of 2 weeks. Following each immunization, immunoglobulin G (IgG) in sera, secretory immunoglobulin A (sIgA) in jejunum lavage, immune-related cytokines, and T-cell proliferation were detected. Following challenge with the highly virulent FAdV-4, the protective effects of the probiotics surface-delivering Fiber2 protein were evaluated by verifying inflammatory factors, viral load, liver function, and survival rate.

**Results:**

The results demonstrated that probiotics surface-delivering Fiber2 protein stimulated humoral and intestinal mucosal immune responses in chickens, shown by high levels of sIgA and IgG antibodies, substantial rise in mRNA levels of cytokines, increased proliferative ability of T cells in peripheral blood, improved liver function, and reduced viral load in liver. Accordingly, adequate protection against homologous challenges and a significant increase in the overall survival rate were observed. Notably, chickens orally immunized with *E. faecalis*/DCpep-Fiber2-CWA were completely protected from the FAdV-4 challenge, which is better than *L. lactis*/DCpep-Fiber2-CWA.

**Conclusion:**

The recombinant probiotics surface-expressing Fiber2 protein could evoke remarkable humoral and cellular immune responses, relieve injury, and functionally damage target organs. The current study indicates a promising method used for preventing FAdV-4 infection in chickens.

## Introduction

Hydropericardium syndrome (HHS), gizzard erosion, and inclusion body hepatitis (IBH) which were previously reported to be associated with fowl adenovirus (FAdV) infection have reemerged in recent years, which has already caused substantial economic losses to the poultry industry worldwide (1, 2). Since 2015, HHS has been commonly reported to be caused by novel fowl adenoviruses serotype 4 (FAdV-4) in China (3–7). The main pathological changes for the reemerged HHS are the accumulation of clear straw-colored fluid in the pericardial sac, synergistic hepatitis, and nephritis (8, 9). HHS is transmitted vertically and horizontally and is characterized by the sudden death of broiler chickens, with mortality rates ranging from 20% to 80% (10, 11). Currently, vaccination remains the most effective measure to prevent and control this infectious disease (12). Routine vaccines have been developed against FAdV-4 infection, including formalin-inactivated infected liver homogenates and inactivated or live attenuated vaccines (13). However, there are still many risks of active infection for applying live, attenuated, and even inactivated vaccines (14). Furthermore, vaccines that effectively stimulate intestinal mucosal immunity are not commercially available. Thus, there is a need for exploring novel vaccines. Mucosal immunity is the first line of defense against pathogenic microorganisms in the intestine, where most immunoglobulin-producing cells are concentrated (15, 16). In recent years, the development of intestinal mucosal vaccines has been increasingly explored. Generally, vaccination aims to generate immunological memory that responds faster upon reexposure to the pathogen before disease symptoms appear (17). Nevertheless, secretory IgA (sIgA) is not only an essential effector molecule for intestinal mucosal immune responses but also associated with immune memory.

Lactic acid bacteria (LAB) are becoming attractive for use as a food additive and vaccine development platform for its classification as generally recognized as safe (GRAS) (18–20). Increasing investigations have shown that probiotics that deliver antigens of zoonotic pathogens, for example, the circumsporozoite protein of *Plasmodium falciparum* delivered by *L. lactis MG1363* (21) and the spike (S) protein receptor-binding domain (RBD) S1 subunit of SARS-CoV-2 delivered by *Lactococcus lactis* IL1403 (22), induced strong mucosal immune responses. Likewise, probiotics *L. plantarum* that display avian pathogen antigenic proteins, such as gp85 of subgroup J avian leukosis virus (ALV-J) (23), *L. lactis* that express ORF2 of avian hepatitis E virus (aHEV) (24), *E. faecalis* that express 3-1E, and *L. plantarum* that deliver MIC2 of *Eimeria tenella* (25, 26), have shown protective efficacies against poultry diseases. Meanwhile, our previous studies demonstrated that intestinal mucosal and humoral immune responses elicited by oral immunization with a probiotic vaccine expressing ΔHexon protein provided partial protection against FAdV-4 (27). FAdV-4 has a typical structure that consists of four structural proteins, namely, Hexon, Penton, Fiber1, and Fiber2 (28). Several latest studies have shown that the pathogenicity of China’s newly emerged highly pathogenic FAdV-4 is closely related to the Fiber2 gene (29–31). The Fiber2 protein-based subunit vaccine offered protection against the FAdV-4 challenge (32).

Given the high pathogenicity of FAdV-4 and the lack of intestinal mucosal immune activation stimulated by traditional vaccines, the development of probiotic vaccines delivering Fiber2 and its role in fighting against FAdV-4 infection are worth exploring. Dendritic cells (DCs) are known to be the primary antigen-presenting cells (APCs) and play an essential role in modulating innate and adaptive immunity (33). After contacting antigens, DCs take up antigens and deliver them to immune effector cells to generate an immune response (34). Previous findings demonstrated that lactic acid bacteria (LAB) delivering DC-targeting peptides (DCpep) enhanced antigen-specific sIgA production by activating the immune responses of DCs (35–37). Hence, in the present study, the fusion protein DCpe-Fiber2 was displayed on the surface of *L. lactis* NZ9000 and *E. faecalis* MDXEF-1 to enhance the Fiber2-specific intestinal mucosal and adaptive immune responses in chickens by targeting DCs, and also the antiviral effects provided by the two live recombinant bacteria were evaluated.

## Materials and Methods

### Bacteria, Virus, and Animals

Recombinant Fiber2 (rFiber2) protein was expressed in *Escherichia coli* (DE3) and purified with BeyoGold™ His-tag Purification Resin (Beyotime, Shanghai). Preparation of ant-rFiber2 polyclonal antibody was performed as described in the previous report (38). rFiber2 protein and ant-rFiber2 polyclonal antibody were characterized and then stored in our laboratory till use. Bacterial strains *L. lactis* NZ9000, *E. faecalis* MDXEF-1, and modified strains were cultured in M17 broth (Luqiao, Beijing, China) with 0.5% glucose (GM17) or GM17 plates at 30°C without shaking (25). Details of the used bacterial strains and plasmids are given in **Table S1**. A chicken hepatoma cell line (LMH) was cultured in a DMEM medium with 10% final fetal bovine serum (Biological Industries, Beit HaEmek, Israel) at 37°C and 5% CO_2_. FAdV-4 strain GS01 (FAdV-4/GS01), a highly virulent strain isolated from natural cases in a chicken farm in Gansu Province, was characterized, purified, and propagated in LMH cells. The specific pathogen-free (SPF) chickens and fertilized SPF chicken eggs were purchased from Harbin Veterinary Research Institute (Heilongjiang, China). Serial 10-fold dilutions of FAdV-4/GS01 were used to infect LMH cells by incubating at 37°C for 3 days to determine TCID_50_ (50% tissue culture infective dose). Then, the formula described by Reed and Muench (39) was applied to calculate it. All chickens were fed with complete formula feed and free access to drinking water in a 12-h light–dark circle environment. Animal experiments were performed according to Ethics Committee for Animal Sciences regulations at Northeast Agricultural University, Heilongjiang Province, China (NEAUEC20210332).

### Preparation of Recombinant Probiotics

**Figure 1:**
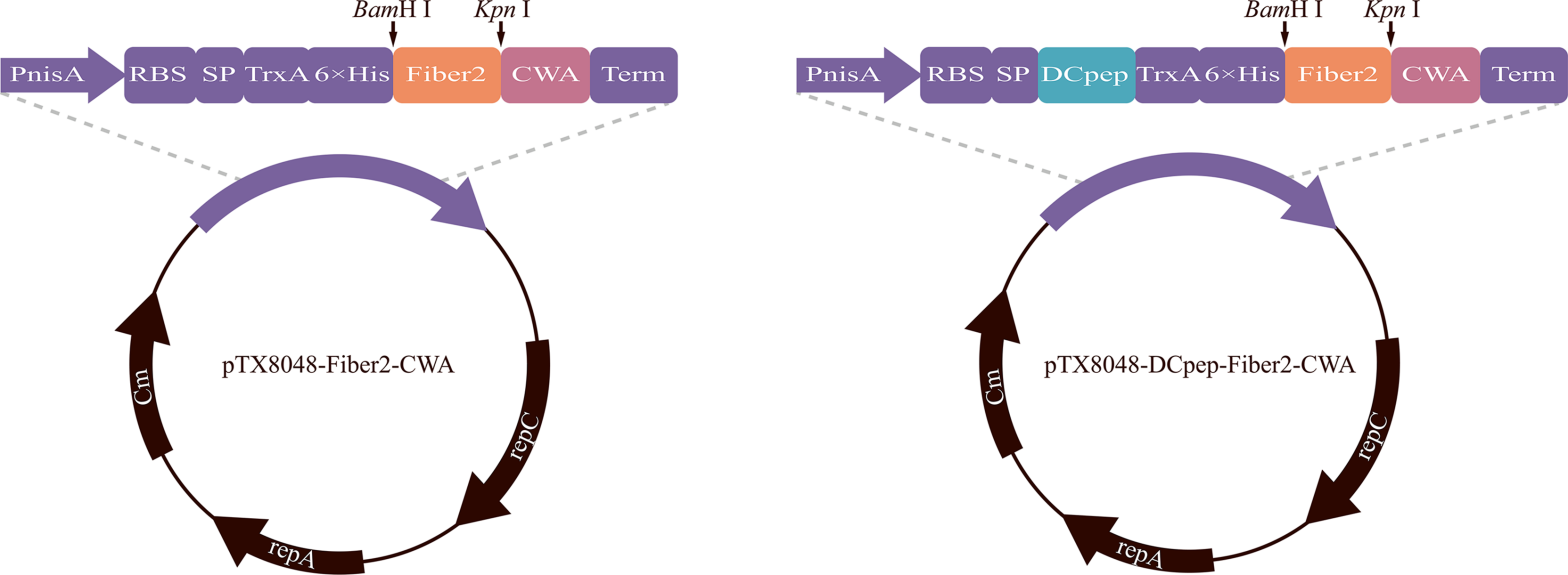
Plasmid schematic of pTX8048-Fiber2-CWA and pTX8048-DCpep-Fiber2-CWA. Both plasmids contain the nisin-inducible promoter and CWA (cell wall anchor) that was fused to the C-terminal of Fiber2 protein. Plasmids pTX8048-DCpep-Fiber2-CWA contained a fused dendritic cell targeting peptide (DCpep) at the N-terminal of Fiber2.

The gene encoding Fiber2 protein was synthesized according to codons optimized for lactic acid bacteria and subcloned into pUC57 by Sangon Biotech Co., Ltd. (Shanghai, China). Plasmid pUC57-Fiber2, pTX8048-CWA (surface-anchoring expression vector) (40), and pTX8048-DCpep-CWA plasmid (surface-anchoring expression vector, fusion with DCpep) (40) were digested with restriction enzymes *Bam*H I and *Kpn* I (TaKaRa, Dalian, China) to release the target Fiber 2 fragment and linearized vector. After ligation using T4 ligase (TaKaRa, Dalian, China), the gene fragment Fiber2 was inserted into the corresponding sites in pTX8048-CWA or pTX8048-DCpep-CWA to produce plasmid pTX8048-Fiber2-CWA and pTX8048-DCpep-Fiber2-CWA (**Figure 1**). Plasmids pTX8048-CWA and pTX8048-DCpep-CWA were designed as control. The two plasmids, pTX8048-Fiber2-CWA and pTX8048-DCpep-Fiber2-CWA, were characterized by sequencing and then electrotransformed (2,000 V, 400 Ω, 25 μF) into host strains *L. lactis* NZ9000 and *E. faecalis* MDXEF-1, respectively. The recombinant bacteria were screened on the plate containing chloramphenicol with a final concentration of 10 μg/ml. After identification by restriction enzyme digestion, the positive recombinant bacteria were named *L. lactis*/pTX8048-Fiber2-CWA, *L. lactis*/pTX8048-DCpep-Fiber2-CWA, *E. faecalis*/pTX8048-Fiber2-CWA, and *E. faecalis*/pTX8048-DCpep-Fiber2-CWA, respectively.

### Detection of Target Proteins

Four recombinant DNA-positive bacteria were seeded on the plate, and the single colony was transferred to a liquid medium. When the value of OD_600_ reached 0.5, 5 ng/ml of nisin (Sigma-Aldrich, USA) was added into the medium for induction for 4 h. Bacterial proteins were prepared according to procedures previously developed in our laboratory (40). Ten percent of SDS-PAGE was applied to separate the bacterial proteins to confirm the expression of the Fiber2 protein in recombinant positive bacteria. The target protein was then electrophoretically transferred to nitrocellulose membranes. The membranes were incubated with rabbit anti-rFiber2 polyclonal antisera (1:2,000, prepared in our laboratory) for 2 h, and horseradish peroxidase (HRP)-labeled goat anti-rabbit IgG antibody (1:2,500) (Sigma, USA) was used as a secondary antibody. After washing, the immunoreactive bands on the membrane were visualized using ECL Chemiluminescence Detection Kit (P0018S) (Beyotime, Shanghai, China) according to the manufacturer’s instructions. To further demonstrate that Fiber2 protein was displayed on the surface of recombinant probiotics, an indirect immunofluorescence assay (IFA) was carried out as described previously (27). In short, the probiotics were cultured and induced with nisin (5 ng/ml), and then the bacterial pellets were harvested by centrifugation. Following three washes with PBS (pH 7.2), the pellets were incubated with rabbit anti-rFiber2 polyclonal sera (1:200) and then with fluorescein isothiocyanate (FITC)-conjugated goat anti-rabbit IgG (1:50) (Solarbio, Beijing, China). The fluorescence images were obtained with a Leica DM2000 fluorescence microscope (Leica, Germany).

### Immunization and Challenge Experiment

**Figure 2:**
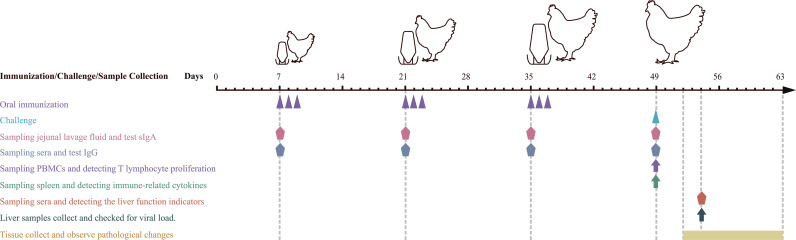
Schematic diagram of immune, sampling, and challenge procedure. Oral immunization with recombinant probiotics *L. lactis* and *E. faecalis* at 7, 8, and 9, 21, 22, and 23, 35, 36, and 37 days of age. Infection experiments were carried out at 49 days of age. Sera and jejunal lavage fluid samples were collected at 7, 21, 35 (before vaccination), and 49 (before challenging). Sera from chickens in each group were collected on day 5 postinfection (dpi) to detect the liver function indicators. Liver samples were collected at 5 dpi to detect inflammatory factors and viral load. Euthanasia and autopsy were performed on 3 dpi to observe gross pathological lesions and histopathological changes.

Grouping, immunizations, and challenges were carried out according to the diagram shown in **Figure 2**. In short, 220 SPF chickens were randomly divided into negative control, *L. lactis*/pTX8048, *E. faecalis*/pTX8048, *L. lactis*/pTX8048-Fiber2-CWA, *L. lactis*/pTX8048-DCpep-Fiber2-CWA, *E. faecalis*/pTX8048-Fiber2-CWA, and *E. faecalis*/pTX8048-DCpep-Fiber2-CWA, and infection control groups for a total of eight groups. All chickens were orally gavaged with recombinant bacteria thrice 2 weeks apart, 3 days in a row (**Table 1**). Samples (sera, liver, and jejunal lavage fluid) collected from chickens (n = 5) in each group at each time point were stored at -80℃ before assays **(** **Figure 2** **)**. All groups (except the negative control group) were challenged with 200 µl 10^5.52^ TCID_50_ FAdV-4/GS01 (stored in our lab) (**Table 1** **).**

**Table 1:** Experimental design of immunizations and challenge.

Groups	Numbers of chicken	Immunizations at days of age			Challenge at days of age
		Primary	Secondary	Third	
		7, 8, and 9	21, 22, and 23	35, 36, and 37	49
Negative control	40	PBS (pH 7.2)			/
*L. lactis*/pTX8048	30	*L. lactis* NZ9000/pTX8048, 1.0 × 10^10^CFU			10^5.52^ TCID_50_
*E. faecalis*/pTX8048	30	*E. faecalis* MDXEF-1/pTX8048, 5.0 × 10^9^CFU			10^5.52^ TCID_50_
*L. lactis*/pTX8048- Fiber2-CWA	30	*L. lactis* NZ9000/pTX8048-Fiber2-CWA, 1.0 × 10^10^CFU			10^5.52^ TCID_50_
*L. lactis*/pTX8048-DCpep-Fiber2-CWA	30	*L. lactis* NZ9000/pTX8048-DCpep-Fiber2-CWA, 1.0 × 10^10^CFU			10^5.52^ TCID_50_
*E. faecalis*/pTX8048- Fiber2-CWA	30	*E. faecalis* MDXEF-1/pTX8048-Fiber2-CWA, 5.0 × 10^9^ CFU			10^5.52^ TCID_50_
*E. faecalis*/pTX8048-DCpep-Fiber2-CWA	30	*E. faecalis* MDXEF-1/pTX8048-DCpep-Fiber2-CWA, 5.0 × 1.0 × 10^9^ CFU			10^5.52^ TCID_50_
Infection Control	10	PBS (pH 7.2)			10^5.52^ TCID_50_

At 49 days of age, 10 chickens were randomly selected from the negative control group to form the infection control group before challenge.

### Detection of IgG and sIgA

Sera and jejunal lavage fluid were prepared at weeks 1, 3, 5, and 7 (before immunizations), as shown in **Figure 2**, and used to detect levels of Fiber2-specific sIgA or IgG antibodies using enzyme-linked immunosorbent assay (ELISA). Briefly, 96-well plates were coated overnight at 4°C with purified 10 μg of rFiber2 proteins (200 μl) as mentioned above. Next, the plates were washed three times with PBST (PBS containing 1% Tween-20) and then saturated with a 100-μl blocking solution that consists of PBS (pH7.2) and 2% BSA at 37°C for 2 h. The sera (1:100 dilution) and jejunal lavage fluid (1:50 dilution) were used as the primary antibody, and HRP-conjugated goat anti-chicken IgG or IgA (Abcam, Cambridge, UK) was used as the secondary antibody, respectively. The reaction was terminated by adding 50 μl of H_2_SO_4_ at a final concentration of 2 M to each well followed by color development by using tetramethylbenzidine (TMB) (Sigma-Aldrich, St. Louis, MO, USA) as the substrate. The absorbance was then measured at 450 nm.

### Measurement of Cytokine Levels After Immunizations

At 2 weeks after the third immunization, the mRNA levels of cytokines in spleen tissues (n = 5) from each group, including chicken interleukin 2 (ChIL-2), ChIL-4, ChIL-6, ChIL-10, ChIL-17, chicken interferon-gamma (ChIFN-γ), and housekeeping gene β-actin, were quantified using real-time PCR, and primers used for real-time PCR are shown in **Table S2**. Total RNA was extracted by applying TRIzol reagent (Invitrogen, Carlsbad, CA, USA) following the manufacturer’s instructions and was then subjected to reverse transcription by using the PrimeScript RT Kit (RR037A) (TaKaRa, Dalian, China). The ΔΔ-Ct method was used to assess levels of gene expression by using SYBR Premix Ex Taq II Reagent Kit (TB Green^®^ Premix Ex Taq™ II) (RR820A) (Takara, Dalian, China).

### Measurement of T-Cell Proliferation After Immunizations

At 2 weeks after the final vaccination, the proliferative activity of peripheral blood lymphocytes (PBMCs) was assayed using the Cell Counting Kit-8 (CCK-8) method (41). PBMCs were obtained using a peripheral blood mononuclear cell isolate kit (P5250) from Beijing Solarbio Science & Technology Co., Ltd. (Beijing, China). PBMCs prepared from chickens (n = 5) in each group were harvested and diluted to a final concentration of 1.0 × 10^6^ cells/ml with RPMI 1640 (containing 10% fetal bovine serum). Then, 2.0 × 10^5^ cells (200 µl) were transferred to a 96-well culture plate, and ConA (10 µg/ml) (Biotopped, Shanghai, China) and rFiber2 proteins (25 µg/ml) were added and incubated at 37°C for 48 h, respectively. Subsequently, 10 μl of CCK-8 solution (Bimake, Houston, TX, USA) was added and incubated for another 4 h, and the value of OD_450 nm_ in each well was recorded. Each sample was tested in triplicate.

### Detection of Hepatic Function

According to the manufacturer’s protocol, the serum activities of alanine transaminase (ALT), aspartate aminotransferase (AST), lactate dehydrogenase (LDH), albumin (ALB), and total protein (TP) were measured at 5 days postinfection (dpi) using commercialized kits purchased from Nanjing Jiancheng Biological Engineering Institute (Nanjing, China).

### Detection of Viral Load in the Liver

At 5 dpi, total DNA was directly extracted from liver samples (n = 5) in each group using TaKaRa MiniBEST Viral RNA/DNA Extraction Kit Ver.5.0 (Takara, Beijing, China). FAdV DNA in liver samples was detected using SYBR Green reagent by real-time PCR as previously described (42). The primer pairs 52K-F/52K-R used are listed in **Table S2**. Furthermore, protein extraction of liver samples was performed using precooled RIPA lysis buffer containing protease inhibitors and PMSF (Solarbio, Beijing, China).

### Detection of Inflammatory Factors Postinfection

The levels of inflammatory factors IL-1β, IL-6, IL-8, and TNF-α in the liver tissues of chickens (n = 3) from each group were detected using ELISA Kit from Shanghai Enzyme-linked Biotechnology Co., Ltd. (Shanghai, China), according to the provided instructions at 5 dpi. The cytokine concentration was calculated based on the constructed standard curve.

### Gross Pathological Changes and Histopathology

Body weights of chickens (n = 10) from each group were recorded at each time point, and body weight gain was calculated. From 3 to 14 dpi, all the chickens from each group were euthanized and autopsied in succession. Gross pathological lesions present on target organs were observed and recorded. The weight of organs, including hearts, livers, kidneys, and spleens, were weighed, and the percentage of organ weight to body weight was calculated. The tissue samples from each group, including livers, hearts, kidneys, and spleens, were fixed in 4% paraformaldehyde solution for 48 h, paraffin-embedded, and cut in slices (5 μm). The slides were then stained with hematoxylin and eosin (HE) staining solution.

### Statistical Analysis

SPSS26 (SPSS/IBM, Chicago, IL, USA), Prism 9.0 (GraphPad Software, La Jolla, CA, USA), and Origin Pro 8 (OriginLab Corporation, Northampton, MA, USA) were used for one-way ANOVA and Duncan’s multiple-comparison analysis of the data. All data were expressed as mean± standard deviation. Differences were considered to be significant at *p* < 0.05 and highly significant at *p* < 0.01.

## Results

### Expression of Fiber2 Protein in Probiotics *L. lactis* and *E. faecalis*

**Figure 3:**
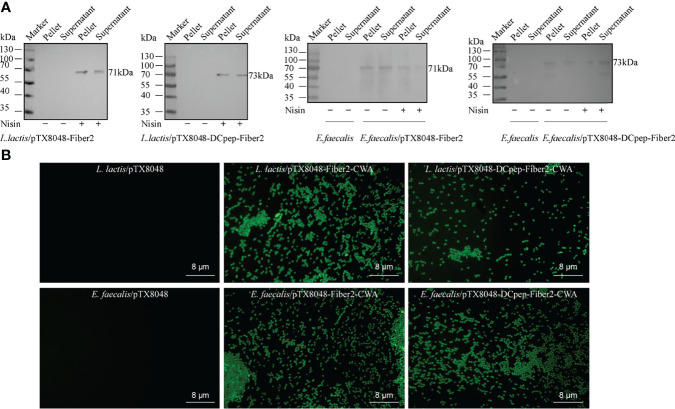
Detection of Fiber2 protein expressed on the surface of probiotics. ***(*A*)*** Western blot detection of Fiber2 protein expression on the surface of probiotics *L. lactis* and *E. faecalis* induced with 5 ng/ml nisin using rabbit anti-Fiber2 polyclonal antisera as primary antibody. **(B)** Indirect immunofluorescence detection of Fiber2 protein displayed on the surface of bacteria using rabbit anti-Fiber2 polyclonal antisera as primary antibody and FITC-conjugated goat anti-rabbit IgG as the secondary antibody.

Recombinant probiotics *L. lactis*/pTX8048-Fiber2-CWA, *L. lactis*/pTX8048-DCpep-Fiber2-CWA, *E. faecalis* MDXEF-1/pTX8048-Fiber2-CWA, and *E. faecalis* MDXEF-1/pTX8048-DCpep-Fiber2-CWA were screened and identified. The cell wall-anchored protein fusion with DC target peptide (DCpep) (73 kDa) or without fusion (71 kDa) in *L. lactis* NZ9000 and *E. faecalis* MDXEF-1 was detected by Western blot (**Figure 3A**). Indirect immunofluorescence experiments also proved that Fiber2 protein was successfully expressed on the surface of the four recombinant probiotics and showed a strong positive fluorescence (**Figure 3B**).

### Fiber2-Expressing Probiotics Induced Humoral Immune Responses

**Figure 4:**
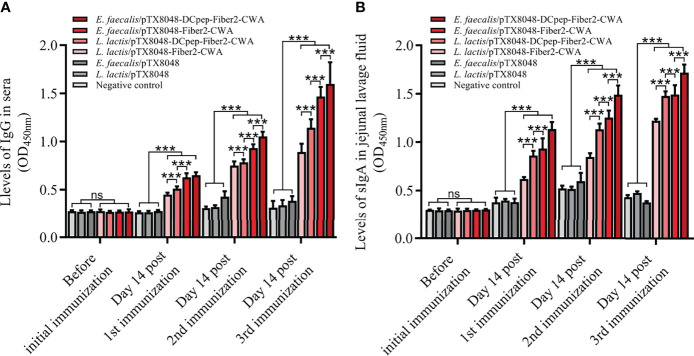
The levels of IgG and sIgA evoked by live recombinant probiotics at each time point. Before the first immunization and at 2 weeks after each immunization, serum samples from chickens (n = 5) in each group were prepared. rFiber2 protein was used to coat the 96-well plate. One hundred-fold diluted experimental chicken sera or 50-fold diluted jejunal lavage fluid was applied as primary antibody to react with coated rFiber2 protein, respectively. The IgG antibody level in sera **(A)** and sIgA antibody level in jejunal lavage fluid **(B)** were analyzed. Data are expressed as mean ± SD. ns means *p* > 0.05, ***p < 0.001.

As shown in **Figure 4**, the levels of serum IgG and jejunal lavage sIgA did not show a statistical difference among the seven groups before the primary immunization (*p* > 0.05). At 2 weeks after the primary, secondary, and third immunizations, the higher IgG and sIgA levels in the four groups with Fiber2-expressing probiotics gradually increased and were all higher than the *L. lactis*/pTX8048, *E. faecalis*/pTX8048, and negative control groups (*p* < 0.01). Fiber2-specific IgG and sIgA in the two DCpep-fusing groups, *L. lactis*/pTX8048-DCpep-Fiber2-CWA and *E. faecalis*/pTX8048-DCpep-Fiber2-CWA, were significantly higher than in other groups (*p* < 0.01). Notably, Fiber2-expressing *E. faecalis*/pTX8048-DCpep-Fiber2-CWA showed the highest humoral immune responses among all the groups (*p* < 0.01). The results demonstrate that probiotics surface-anchoring the target protein and DCpep stimulated more robust humoral immune responses than those expressing a single antigen.

### mRNA Levels of Cytokines in the Spleen After Immunization

**Figure 5:**
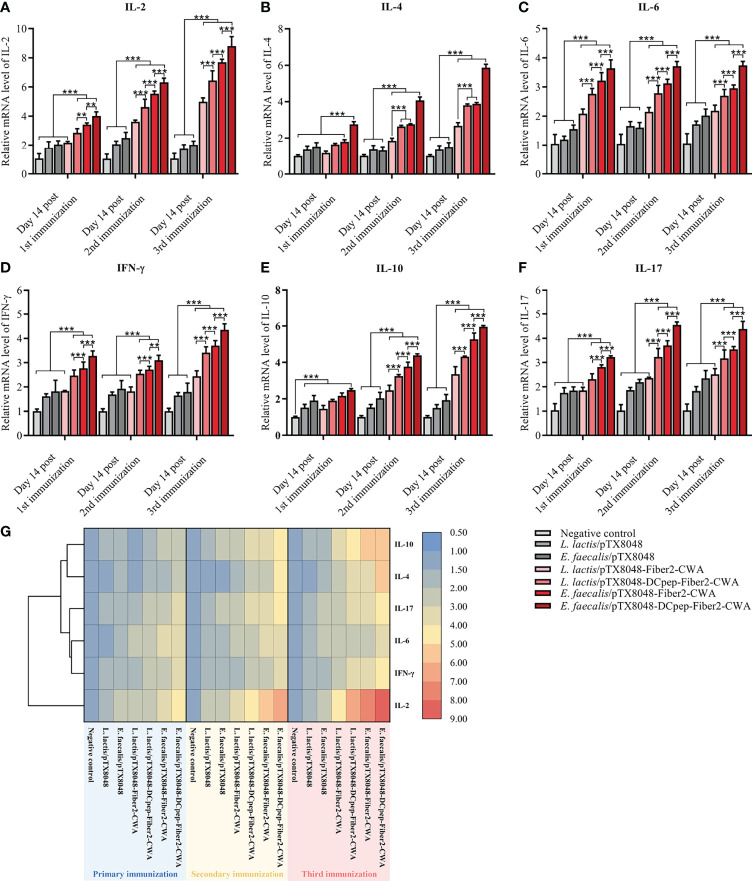
Immune-related cytokine mRNA levels in spleens. The relative mRNA levels of IL-2 **(A)**, IL-4 **(B)**, IL-6 **(C)**, IFN-γ **(D)**, IL-10 **(E)**, and IL-17A **(F)** in spleens of chickens (n = 5) in each group were quantified by real-time PCR and normalized by mRNA levels of reference gene β-actin from the same sample. The values represent mean ± SD. **p < 0.01, ***p < 0.001. **(G)** Heat map depicting the cluster analysis of mean values of ChIL-2, ChIL-4, ChIL-6, ChIFN-γ, ChIL-10, and ChIL-17A in spleens after three immunizations.

Real-time PCR was applied to determine the mRNA levels of cytokines IL-2, IFN-γ, IL-4, IL-10, IL-6, and IL-17 in spleens from immunized chickens. As shown in **Figure 5**, at 2 weeks after the secondary and third immunizations, the four Fiber2-expressing probiotics groups except for *L. lactis*/pTX8048-Fiber2-CWA displayed significantly higher mRNA levels of IL-2, IFN-γ, IL-4, IL-10, IL-6, and IL-17 compared to the *L. lactis*/pTX8048, *E. faecalis*/pTX8048, and negative control groups (*p* < 0.001). Besides, significant differences were also observed among the four Fiber2-expressing probiotics groups, and the mRNA levels of those cytokines in the *E. faecalis*/pTX8048-Fiber2-CWA group and *E. faecalis*/pTX8048-DCpep-Fiber2-CWA groups were higher than those in the *L. lactis*/pTX8048-Fiber2-CWA and *L. lactis*/pTX8048-DCpep-Fiber2-CWA groups, respectively. The above results indicated that Fiber2-expressing *E. faecalis* stimulated higher levels of cytokines than Fiber2-expressing *L. Lactis*. As shown in the cluster heatmap (**Figure 5G**), IL-4 and IL-10 cluster in one branch; IFN-γ, IL-6, and IL-17 cluster in another branch; and IL-2 clusters under one branch alone.

### Proliferation of Lymphocytes in Peripheral Blood

**Figure 6:**
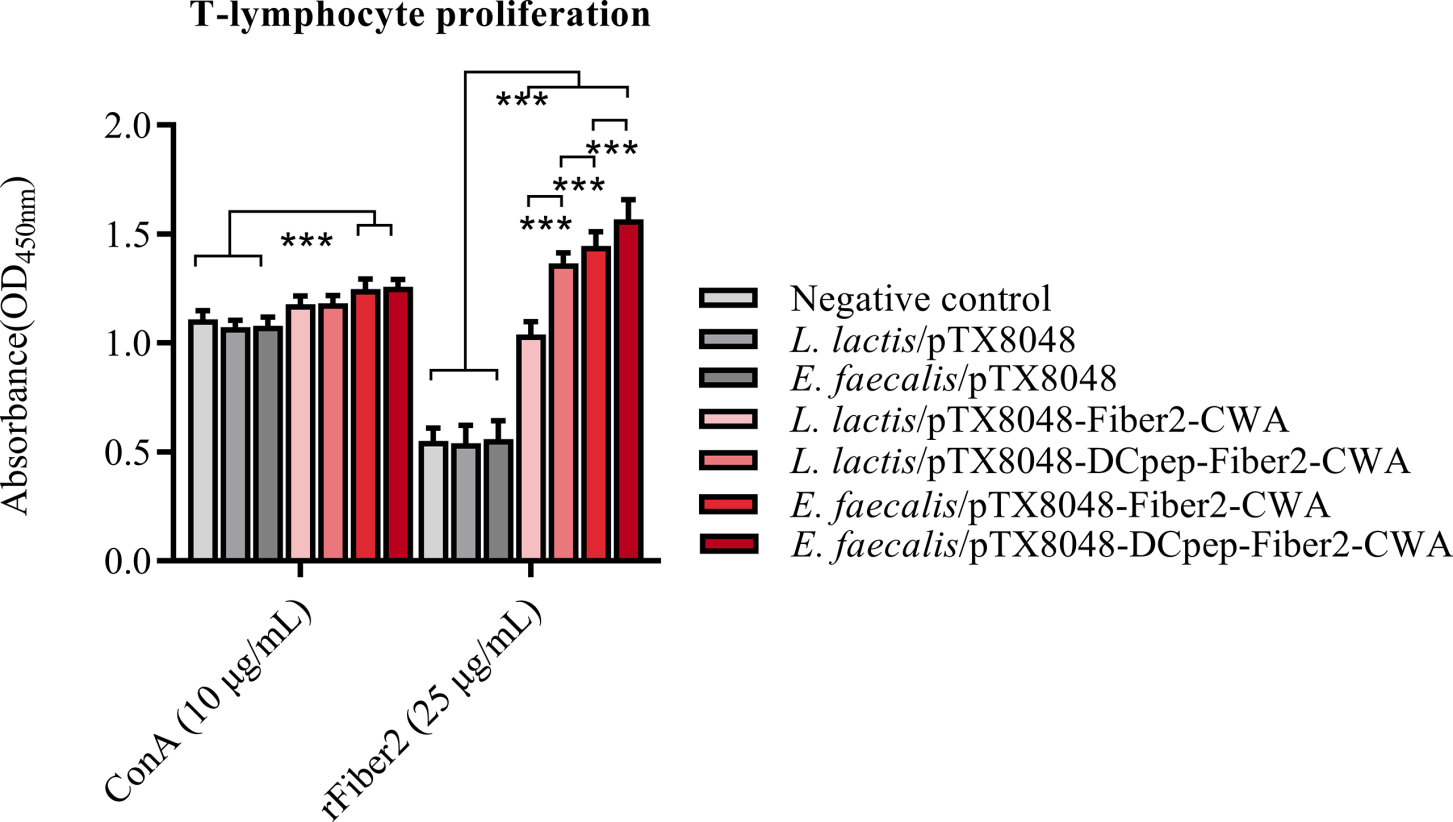
Detection of T lymphocyte proliferation. At 2 weeks after the third immunization, T lymphocytes were isolated from the peripheral blood of five chickens (n = 5) from each group. The proliferation of isolated T lymphocytes was assessed using a CCK-8 assay kit, and rFiber2 protein and ConA were selected as stimuli, respectively. Data are presented as mean ± SD. ***p < 0.001.

At 2 weeks after the third immunization, peripheral blood lymphocytes (PBLs) from chickens immunized with four Fiber2-expressing probiotics showed significant responses to rFiber2 protein compared with the *L. lactis*/pTX8048, *E. faecalis*/pTX8048, and PBS groups (*p* < 0.01). Among the four Fiber2-expressing probiotics, *E. faecalis*/pTX8048-DCpep-Fiber2-CWA displayed the highest capability to induce PBLs to proliferate under stimulation with rFiber2 (*p* < 0.01). Meanwhile, two Fiber2-expressing *E. faecalis* showed a higher capability to induce the proliferative activity of PBLs than the two Fiber2-expressing *L. lactis* under stimulation with ConA (*p* < 0.001, **Figure 6**).

### Levels of Inflammatory Factors in Liver After Challenge

**Figure 7:**
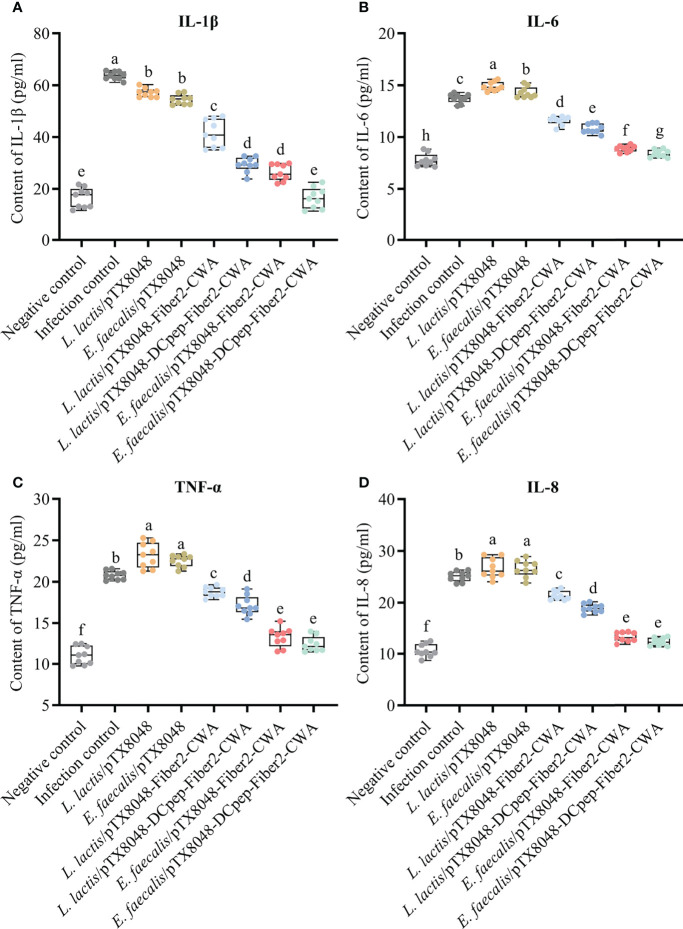
Detection of inflammatory factors in livers of chickens at 5 days postinfection. The levels of inflammatory factors IL-1β **(A)**, IL-6 **(B)**, TNF-α **(C)**, and IL-8 **(D)** in the livers of chickens (n = 3) from each group were determined by ELISA. Each sample was tested in triplicate. Different small letters denote significant differences among groups (*p* < 0.05).

The levels of inflammatory factors in livers were detected to assess the level of inflammation in the livers. The abovementioned inflammatory factors increased dramatically in the *L. lactis*/pTX8048, *E. faecalis*/pTX8048, and infection control groups at 5 dpi compared with the negative control group (**Figure 7**) (*p* < 0.05). On the contrary, the four Fiber2-expressing probiotics groups, especially *E. faecalis*/pTX8048-Fiber2-CWA and *E. faecalis*/pTX8048-DCpep-Fiber2-CWA, increased less (*p* < 0.01 or *p* < 0.001), although significantly higher than the negative control group.

### Detection of Function Indexes

**Figure 8:**
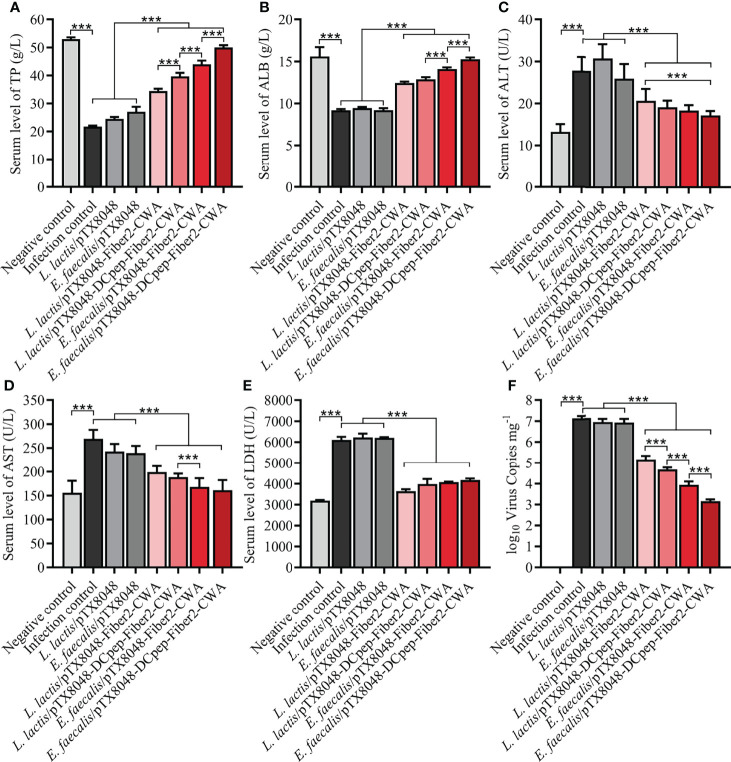
Determination of alanine transaminase (ALT), aspartate aminotransferase (AST), lactate dehydrogenase (LDH), albumin (ALB), and total protein (TP) in sera. At 5 dpi, levels of liver function indexes including TP **(A)**, ALB **(B)**, ALT **(C)**, AST **(D),** and LDH **(E)** in sera of chickens (n = 5) from each group were detected using the purchased kit (Nanjing Jiancheng Bioengineering Institute) referencing provided protocols. At 5 dpi, liver tissues were collected from chickens in each group, and total DNA was extracted using a DNA extraction kit (Takara, Beijing, China). A standard curve was established based on the pMD-18T-52K plasmid. Real-time PCR was used to detect numbers of FAdV DNA copies in livers **(F)**. Each value represents mean ± SD. ***p < 0.001.

The liver function of experimental chickens in each group was evaluated based on widely used biochemical markers TP, ALB, AST, ALT, and LDH. As displayed in **Figures 8A–E**, significant differences were observed between the four Fiber2-expressing probiotics groups and the other four control groups, including infection control, vector control (*L. lactis*/pTX8048, *E. faecalis*/pTX8048), and negative control. Compared with the negative control group, the levels of ALB and TP in sera of chickens in the infection control and vector control groups (*L. lactis*/pTX8048, *E. faecalis*/pTX8048) decreased significantly (*p* < 0.01). In contrast, AST, ALT, and LDH in the four Fiber2-expressing probiotics groups *L. lactis*/pTX8048-Fiber2-CWA, *L. lactis*/pTX8048-DCpep-Fiber2-CWA, *E. faecalis*/pTX8048-Fiber2-CWA, and *E. faecalis*/pTX8048-DCpep-Fiber2-CWA did not change significantly but displayed to be close to the negative control group (*p* < 0.001).

### Virus Load in the Liver

At 5 dpi, the average numbers of FAdV copies in livers of chickens in the infection control group (1.3 × 10^7^ copies/mg), *L. lactis*/pTX8048 group (8.6 × 10^6^ copies/mg), and *E. faecalis*/pTX8048 group (8.2 × 10^6^ copies/mg) were significantly higher than those of chickens immunized with the four Fiber2-expressing probiotics, *L. lactis*/pTX8048-Fiber2-CWA (1.4 × 10^5^ copies/mg), *L. lactis*/pTX8048-DCpep-Fiber2-CWA (4.8 × 10^4^ copies/mg), *E. faecalis*/pTX8048-Fiber2-CWA (9.0 × 10^3^ copies/mg), and *E. faecalis*/pTX8048-DCpep-Fiber2-CWA (1.5 × 10^3^ copies/mg) (*p* < 0.01) (**Figure 8F**). Similar results were also observed in the detection of viral proteins. The content of viral proteins in livers of chickens immunized with the four Fiber2-expressing recombinant lactic acid bacteria was lower than that in the infection control group (**Figure S1**).

### Survival Rate, Body Weight Gain, and Organ Index

**Figure 9:**
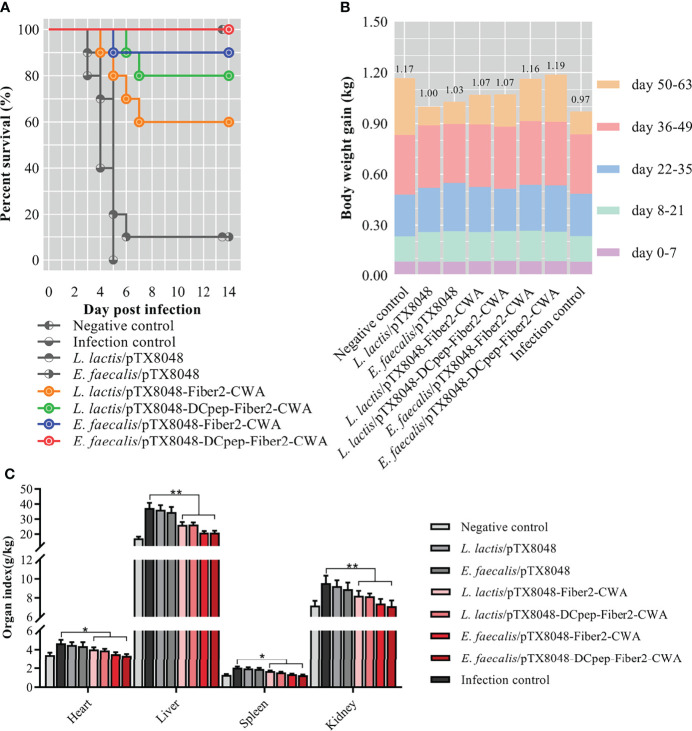
Survival rate, body weight, and relative organ index of chickens. The survival rate **(A)** of chickens in each group was recorded within 2 weeks after the challenge with the highly virulent strain FAdV-4/GS01. Colored lines **(A)** represent groups immunized with live recombinant probiotics. The body weight gain of chickens (n = 10) in each group at each stage was shown by different colors, and the numbers at the top of the columns indicated each final average body weight in each group **(B)**. The organ index of hearts, livers, spleens, and kidneys of chickens from each group was calculated **(C)**. *p < 0.05, **p < 0.01.

On the third day after challenge with FAdV-4/GS01, chickens in the infection control, *L. lactis*/pTX8048, and *E. faecalis*/pTX8048 groups showed clinical signs of depression and achieved 100% mortality at 6 dpi. Chickens in the four groups immunized with Fiber2-expressing probiotics were to some extent protected against FAdV challenge at a 100% lethal dose (**Figure 9A**), displaying the survival rates of 60%, 80%, 90%, and 100% in groups *L. lactis*/pTX8048-Fiber2-CWA, *L. lactis*/pTX8048-DCpep-Fiber2-CWA, *E. faecalis*/pTX8048-Fiber2-CWA, and *E. faecalis*/pTX8048-DCpep-Fiber2-CWA, respectively. Notably, the time of death for the challenged chickens in groups *L. lactis*/pTX8048-Fiber2-CWA, *L. lactis*/pTX8048-DCpep-Fiber2-CWA, *E. faecalis*/pTX8048-Fiber2-CWA, and *E. faecalis*/pTX8048-DCpep-Fiber2-CWA were to some extent delayed compared to the two vector control groups, indicating that the four recombinant live probiotics exhibited more protective efficacy against FAdV-4/GS01 challenge than *L. lactis*/pTX8048 and *E. faecalis*/pTX8048.

Not only is the survival rate an essential criterion for evaluating protection against virus challenge, but also the body weight and relative organ weight are influential factors. Statistics found that the average body weight of chickens in the groups orally immunized with probiotics was higher than that in the infection control group (**Figure 9B**). On the contrary, the organ indexes of hearts, livers, spleens, and kidneys of chickens in groups *L. lactis*/pTX8048-Fiber2-CWA, *L. lactis*/pTX8048-DCpep-Fiber2-CWA, *E. faecalis*/pTX8048-Fiber2-CWA, and *E. faecalis*/pTX8048-DCpep-Fiber2-CWA were significantly lower than those in the infection control group (**Figure 9C**) (p < 0.05).

### Pathological Lesions

**Figure 10:**
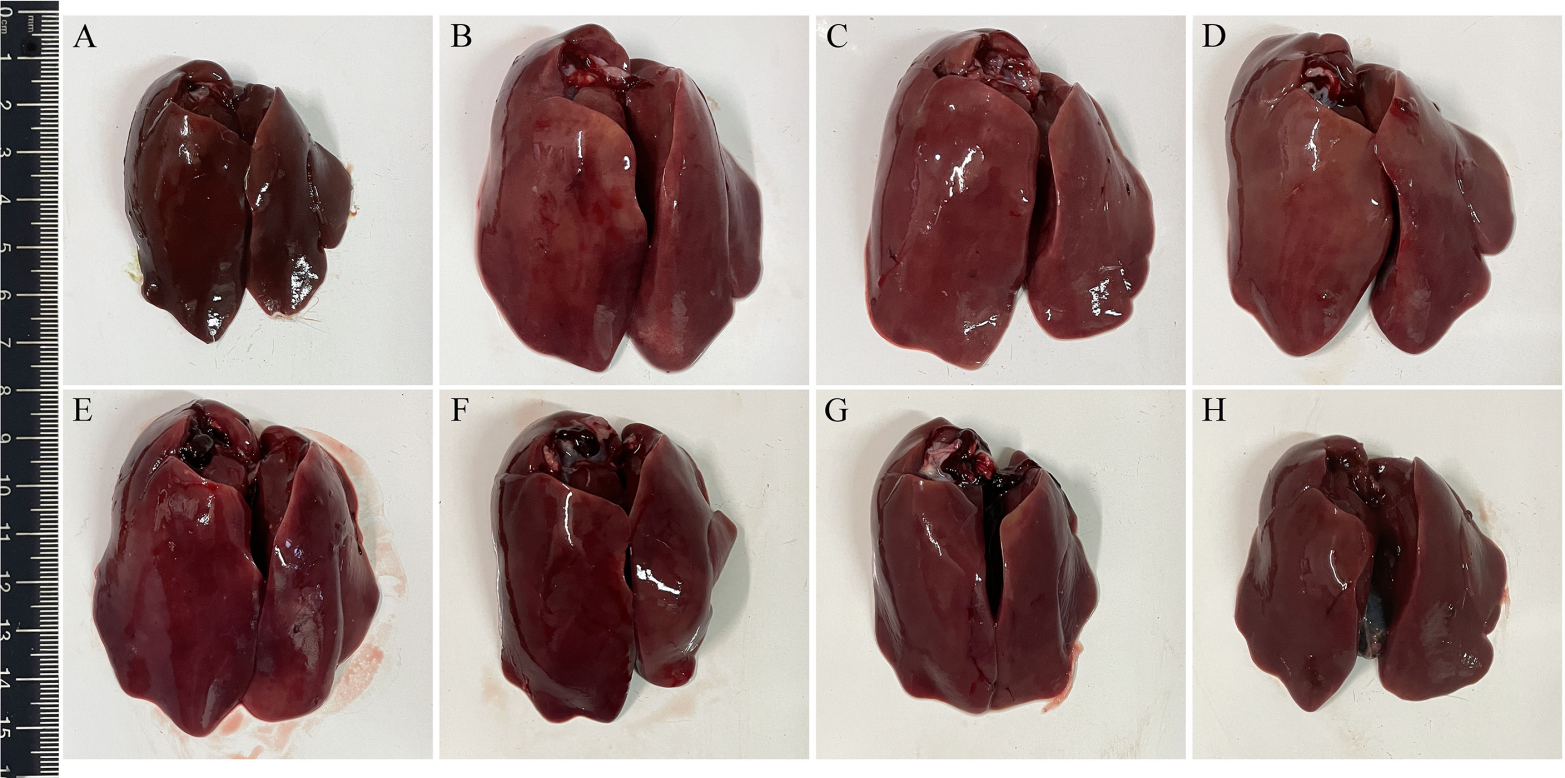
Gross pathological changes in livers of chickens from each group. The livers of chickens in negative control group were normal **(A)**. Pathological changes in infection control **(B)**, *L. lactis*/pTX8048 **(C)**, and *E. faecalis*/pTX8048 **(D)** groups were noticeable, showing swollen and friable, with multifocal areas of necrosis. Only slight pathological lesions were observed in groups *L. lactis*/pTX8048-Fiber2-CWA **(E)**, *L. lactis*/pTX8048-DCpep-Fiber2-CWA **(F)**, *E. faecalis*/pTX8048-Fiber2-CWA, **(G)** and *E. faecalis*/pTX8048-DCpep-Fiber2-CWA **(H)**.

Three days after challenge, typical clinical symptoms including weakness, depression, anorexia, and decreased body weight gain were observed in the infection control group. Accordingly, gross pathological changes found in the infection control group revealed splenomegaly, hepatomegaly, hepatitis, splenitis, and myocarditis. On the contrary, the above symptoms and gross pathological changes in the four groups immunized with *L. lactis*/pTX8048-Fiber2-CWA, *L. lactis*/pTX8048-DCpep-Fiber2-CWA, *E. faecalis*/pTX8048-Fiber2-CWA, and *E. faecalis*/pTX8048-DCpep-Fiber2-CWA were relatively milder. The lesions in the target organ livers are shown in **Figure 10**. Compared with the negative control group, swelling and inflammation in livers of chickens in the *L. lactis*/pTX8048-Fiber2-CWA, *L. lactis*/pTX8048-DCpep-Fiber2-CWA, *E. faecalis*/pTX8048-Fiber2-CWA, and *E. faecalis*/pTX8048-DCpep-Fiber2-CWA groups displayed significant reduction. Also, inflammatory lesions in the hearts and kidneys of chickens in groups *L. lactis*/pTX8048-Fiber2-CWA, *L. lactis*/pTX8048-DCpep-Fiber2-CWA, *E. faecalis*/pTX8048-Fiber2-CWA, and *E. faecalis*/pTX8048-DCpep-Fiber2-CWA were significantly alleviated compared with the infection control group (**Figure S1**).

**Figure 11:**
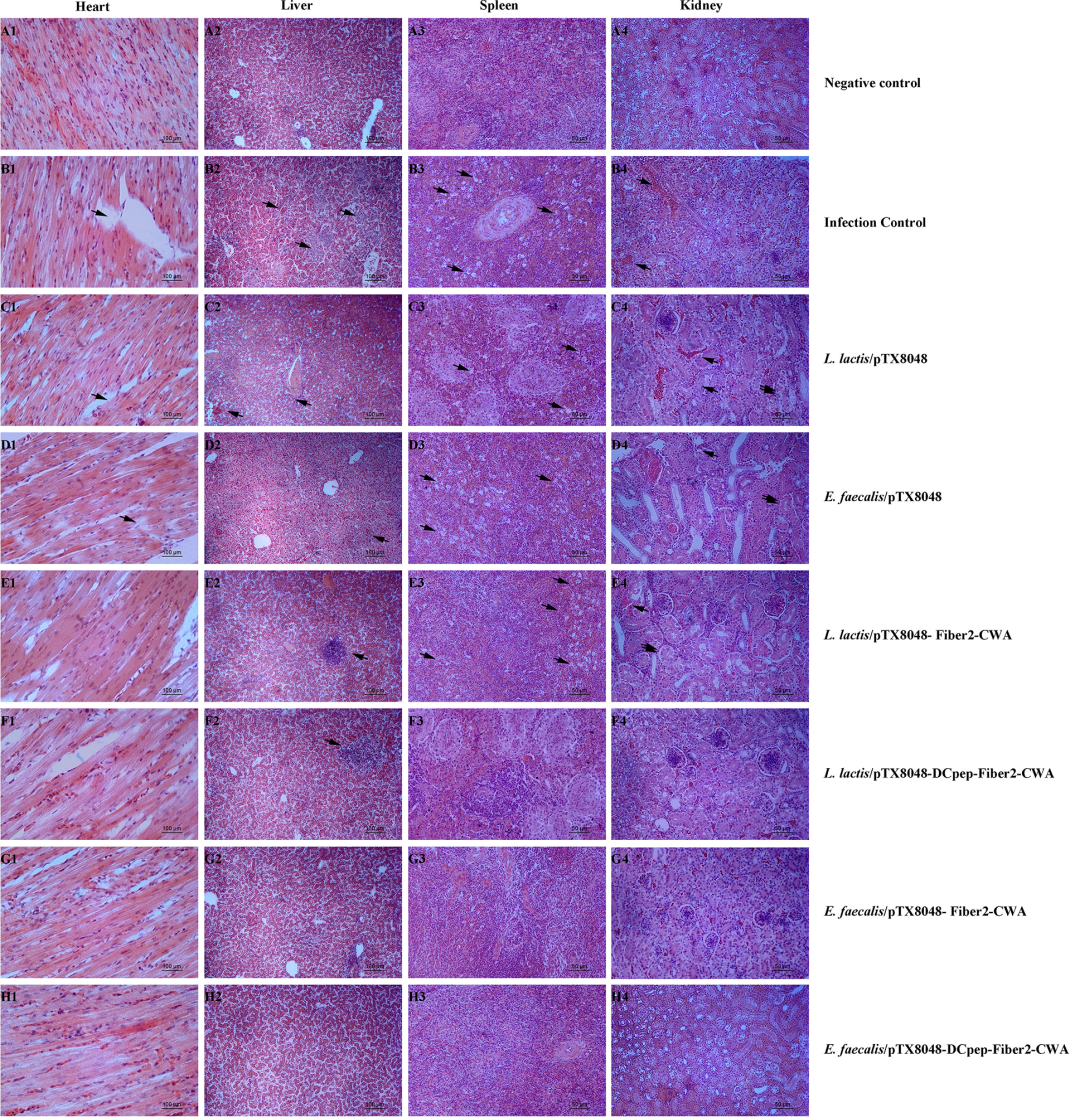
Microscopic histopathological changes in chickens heart, liver, kidney, and spleen tissues from each group. Tissues of hearts, livers, spleens and kidneys from experimental chickens were collected and used to prepare histopathological slides. There are no apparent lesions in the heart, liver, spleen, and kidney in negative control **(A1–A4)**. On the contrary, in infection control, *L. lactis*/pTX8048 and *E. faecalis*/pTX8048 groups showed noticeable lesions. These lesions involve myocardial fiber rupture in cardiac tissue as indicated by arrows, hepatocyte necrosis and inflammatory cell infiltration in liver tissue (arrows), focal necrosis of splenocytes (arrows), tubular epithelial cell shedding, and interstitial congestion (arrows) in renal tissue **(B–D)**. Compared with the control group, the other four challenged groups had relatively fewer lesions **(E–H)**.

On 5 dpi, tissues of hearts, livers, spleens, and kidneys of chickens in each group were fixed in 10% formalin, and histopathological slides were prepared. As shown in **Figure 11**, the noticeable and severe histopathological lesions in tissues of chickens in the infection control group were observed, including myocardial fiber rupture in heart tissues (**Figures 11B1–D1**), vacuolar degeneration and necrosis in liver cells (**Figures 11B2–D2**), necrotic foci and lymphopenia in spleen tissues (**Figures 11B3–D3**), and glomerulonephritis, vasculitis, interstitial congestion, and cortical necrosis in renal tissues (**Figures 11B4–D4**). However, chickens from the four Fiber2-expressing probiotics-vaccinated groups, especially group *E. faecalis*/pTX8048-DCpep-Fiber2-CWA, displayed mild histopathological changes (**Figures 11E–H**).

## Discussion

Hepatitis-hydropericardium syndrome (HHS) is a typical gross pathological change which could be caused by Fowl adenoviruses serotype 4 (FAdV-4). HHS is characterized by a sudden onset and mortality rates ranging from 20% to 80%, which leads to substantial economic losses to the global poultry industry (43). Currently, vaccination is the primary strategy for preventing and controlling HHS. Immunization with inactivated liver homogenate (14, 44), attenuated vaccines (32), live vaccines (45), and recombinant antigen vaccines (46) protects against infection. Inactivated vaccines have a lower risk of virulence reversion than live vaccines. Other research on FAdV also emphasized the risk of incomplete inactivation or reduced efficacy (47, 48). As mentioned before, intramuscular injections of inactivated vaccines or attenuated vaccines failed to activate intestinal mucosal immunity. Recently, subunit vaccines have been reported to be similar to inactivated vaccines and have displayed the characteristics of stability and safety (46, 49). Theoretically, vaccination that evokes effective intestinal mucosal immune responses is a feasible way to prevent pathogens transmitted *via* an oral route (49). Therefore, novel vaccines that can activate intestinal mucosal immunity against FadV-4 infection are still attractive.

Our previous results showed that the oral administration of lactic acid bacteria expressing truncated Hexon proteins could offer protection against FAdV-4 to some extent (26). Recently, Fiber2 protein has been proved to offer better protection than Hexon protein in vaccines prepared using the baculovirus expression system (50) and *E. coli* expression system (51), although this is not a comparison between Fiber2 and intact Hexon protein. The increased virulence of hypervirulent FAdV-4 was reported to be independent of fiber-1 and penton (52). The study on infectious clones of FAdV-4 displayed that fiber 2 protein plays a greater role in FAdV-4 pathogenicity than Hexon protein (29). Moreover, it was reported that adenovirus was located in the intestinal epithelium at 12 h post-oral infection, and the virus was detected in blood at 24 h postinfection (53). Considering the above facts, we hypothesize that Fiber2-specific sIgA secreted on the surface of intestinal epithelium could bind to FAdV-4 at the local sites and hence intercept virus invasion upon initial infection. Therefore, in the current study, probiotics that surface-deliver Fiber2 protein were prepared, and the protective immunity against FAdV-4 infection was evaluated.

The ability to activate an effective immune response is an essential feature of a successful vaccine. The levels of IgG in serum and sIgA in jejunal lavage fluid are an indicator of humoral and intestinal mucosal immunity, respectively. In the present study, the levels of Fiber2-specific IgG in sera and sIgA in the intestinal mucosa were significantly increased, suggesting that the Fiber2 protein delivered in surface-anchoring by the four recombinant probiotics effectively induced humoral and intestinal mucosal immune responses in all immunized chickens and hence provided immune protection. The above results are consistent with the previous report that recombinant subunit vaccines based on Fiber2 protein provided more comprehensive protection against FAdV-4 infection than inactivated oil emulsion vaccines (54).

Moreover, the immune protective effects provided by recombinant probiotics *L. lactis*/pTX8048-DCpep-Fiber2-CWA and *E. faecalis*/pTX8048-DCpep-Fiber2-CWA) were better than those by *L. lactis*/pTX8048-Fiber2-CWA and *E. faecalis*/pTX8048-Fiber2-CWA, respectively (**Figure 4**). A possible derivation for these results is that dendritic cells in the intestinal lamina propria were effectively activated by DCpep, which enhanced the antigenic uptake and the subsequent delivery to immune cells. The immune enhancer DCpep is unanimous to other previous reports (25, 55). In addition, recombinant live bacteria *E. faecalis*/pTX8048-Fiber2-CWA and *E. faecalis*/pTX8048-DCpep-Fiber2-CWA induced more obvious immune responses and provided more protective effects than *L. lactis*/pTX8048-Fiber2-CWA and *L. lactis*/pTX8048-DCpep-Fiber2-CWA (*p* < 0.05), respectively. The possible explanation for this result is that recombinant bacteria *E. faecalis*/pTX8048-Fiber2-CWA and *E. faecalis*/pTX8048-DCpep-Fiber2-CWA sustainably evoked Fiber2-specific immune responses. The supported findings for the above explanation are that *E. faecalis*/pTX8048-Fiber2-CWA and *E. faecalis*/pTX8048-DCpep-Fiber2-CWA can express the Fiber2 protein on the surface of probiotic bacteria without nisin induction (**Figure 2A**). The potential reason for this is that *E. faecalis* MDXEF-1 produces nisin or similar ingredients (data not published), which act as an inducer to initiate transcription and translation of the target gene located downstream of the nisA promoter in the positive plasmids.

The previous reports demonstrated that cellular immunity played an important role in antiviral immunity, especially in preventing FAdV-4, a virus with immunosuppressive potential (56). ChIL-2 and ChIFN-γ are generally produced by Th1 cells and have multiple functions (57). The above two cytokines activate transcription factors that regulate the production of several defense molecules by natural killer cells, cytotoxic T cells, and macrophages (58–60). In addition, IFN-γ cooperates with other factors, such as ChIL-17, to activate macrophages to kill phagocytized pathogens and infected cells (61) and promote immunoglobulin production. ChIFN-γ also effectively induces, maintains, and links innate immunity to adaptive immune responses (62). In the present study, the proliferative responses of PBLs were more efficient in the groups immunized with Fiber2-expressing probiotics (**Figure 6**). Moreover, the mRNA levels of ChIL-2, ChIFN-γ, ChIL-4, ChIL-10, ChIL-6, and ChIL-17 in spleens of chickens immunized with the four Fiber2-expressing probiotics were significantly higher, which suggests that Th10-, Th2-, and Th17-type responses contribute to the strengthened immunity and resistance to virus infection (**Figure 5**). Previous studies also reported similar results showing that the expression of several inflammatory factors such as ChIFN-γ, ChIL-2, ChIL-4, ChIL-6, and ChIL-10 increased during FAdV-4 infection (63, 64). The above results could be supported by the following analysis that on 5 dpi, the number of FadV copies in the livers of chickens from the four groups immunized with *L. lactis*/pTX8048-Fiber2-CWA, *L. lactis*/pTX8048-Dcpep-Fiber2-CWA, *E. faecalis*/pTX8048-Fiber2-CWA, and *E. faecalis*/pTX8048-Dcpep-Fiber2-CWA, especially the two groups of Fiber2-expressing *E. faecalis*, was significantly lower than that in the two groups immunized with *L. lactis*/pTX8048 and *E. faecalis*/pTX8048 (**Figure 8F**). The necrotic foci in the spleens from the two Fiber2-expressing *E. faecalis* groups were significantly reduced after the challenge (**Figure 10** **A3-H3**), which also supports our previous analysis. Collectively, all these results suggest that Fiber2-expressing probiotics *L. lactis* and *E. faecalis* could stimulate robust humoral and cellular immune responses which activate antiviral protection by moderately enhancing the expression of inflammatory factors.

The liver is generally accepted to be the primary target organ for FAdV-4. The liver injury caused by FAdV-4 could be ascribed to the increased permeability of the hepatocyte membrane, which leads to the release of the intracellular substances into the blood, therefore resulting in a dramatic increase in ALT and AST (65). In this study, AST, ALT, and LDH levels in sera in the probiotic-treated groups, including *L. lactis*/pTX8048-Fiber2-CWA, *L. lactis*/pTX8048-DCpep-Fiber2-CWA, *E. faecalis*/pTX8048-Fiber2-CWA, and *E. faecalis*/pTX8048-DCpep-Fiber2-CWA, were significantly lower than in the infection control group, which suggested that probiotics surface-expressing Fiber2 protein can alleviate the liver injury to some extent. The decreased levels of TP and ALB in sera indirectly reflected the diminished FAdV-4 synthesis in the target organ livers (66). Moreover, the slightly changed TP and ALB in the four groups immunized with Fiber2-expressing probiotics also indicated the protective effects against FAdV infection.

It has been reported that the critical inflammatory factors IL-1β, IL-6, IL-8, and TNF-α were significantly increased after FAdV infection (67). In the present study, as shown in **Figure 7**, a significant reduction of IL-1β, IL-6, IL-8, and TNF-α in the groups immunized with *L. lactis*/pTX8048-Fiber2-CWA, *L. lactis*/pTX8048-DCpep-Fiber2-CWA, *E. faecalis*/pTX8048-Fiber2-CWA, and *E. faecalis*/pTX8048-DCpep-Fiber2-CWA was observed compared with the infection control group. Accordingly, the typical gross pathological and histopathological changes were observed in the infection control group, *L. lactis*/pTX8048, and *E. faecalis*/pTX8048, but not in the groups immunized with the four Fiber2-expressing probiotics. The above results indicated that the virus load in livers of chickens vaccinated with Fiber2-expressing *L. lactis* or *E. faecalis* was significantly reduced.

The survival rate is considered to be a central factor in measuring vaccine efficacy. The present results of survival rate showed that chickens immunized with *E. faecalis*/pTX8048-DCpep-Fiber2-CWA exhibited a 100% protection rate. At the same time, the protection rates for live bacteria *L. lactis*/pTX8048-Fiber2-CWA, *L. lactis*/pTX8048-DCpep-Fiber2-CWA, and *E. faecalis*/pTX8048-Fiber2-CWA were 60%, 80%, and 90%, respectively. The above results strongly indicated that the live recombinant probiotics that deliver surface-anchored Fiber2 protein showed excellent protective efficacy. In addition, in poultry farming, body weight gain and daily weight gain are both vital factors from an economic point of view. The body weights of chickens from the four Fiber2-expressing probiotics groups were all higher than those in the infection control group. The above results indicated that different Fiber2-delivering probiotics provided different immune protective effects and survival rates.

## Conclusion

This study shows that our experiments validated the initial assumption and achieved the desired results showing that recombinant probiotics surface-expressing the Fiber2 protein could evoke remarkable humoral and cellular immune responses, relieve injury, and functionally damage target organs. This knowledge has provided a valuable reference for developing potential vaccines to protect against FAdV-4 infection.

## Data Availability Statement

The raw data supporting the conclusions of this article will be made available by the authors, without undue reservation.

## Ethics Statement

The animal study was reviewed and approved by Ethics Committee for Animal Sciences regulations at Northeast Agricultural University, Heilongjiang Province, China. Written informed consent was obtained from the owners for the participation of their animals in this study.

## Author Contributions

DM, ZJ, and CM designed the study. XP, WZ, HC, ZJ, and BB prepared the experimental materials. CM and XP contributed to the analytic tools. ZJ, CM, and DM analyzed the data. ZJ and CM wrote the paper. ZJ, CM, and DM revised the manuscript. All authors contributed to the article and approved the submitted version.

## Funding

This work was supported by the National Natural Science Foundation of China (30901061, 31973003).

## Conflict of Interest

The authors declare that the research was conducted in the absence of any commercial or financial relationships that could be construed as a potential conflict of interest.

## Publisher’s Note

All claims expressed in this article are solely those of the authors and do not necessarily represent those of their affiliated organizations, or those of the publisher, the editors and the reviewers. Any product that may be evaluated in this article, or claim that may be made by its manufacturer, is not guaranteed or endorsed by the publisher.

## Supplementary Material

Supplementary Figure 1 Western blot detection of viral proteins. Detection of viral protein in liver tissues from each group on the 5dpi. Fiber2 polyclonal antibody (1:2000) and HRP-labeled goat anti-rabbit secondary antibody (1:2000) were used for detection. β-actin was used as an internal reference for detection. The amount of viral protein in the four recombinant lactic acid bacteria immunization groups was lower than that in the control group.

Supplementary Figure 2 Gross examination of heart, spleen, and kidney from chickens. The gross pathological changes in infection control, *L. lactis*/pTX8048, and *E. faecalis*/pTX8048Groups were obvious. Their heart and kidneys were significantly enlarged, and the spleen had congestion and enlargement simultaneously. The pathological lesions in groups *L. lactis*/pTX8048-Fiber2-CWA, *L. lactis*/pTX8048-DCpep-Fiber2-CWA, *E. faecalis*/pTX8048-Fiber2-CWA and *E. faecalis*/pTX8048-DCpep-Fiber2-CWA were relatively mild.
