# Supplementary material for: Probiotics Surface-Delivering Fiber2 Protein of Fowl Adenovirus 4 Stimulate Protective Immunity Against Hepatitis-Hydropericardium Syndrome in Chickens
Source: Front Immunol. 2022 Jun 28;13:919100. doi: 10.3389/fimmu.2022.919100 (PMC9273852; doi:10.3389/fimmu.2022.919100)

Supplementary Material

# Supplementary Tables

Table S1 Strains and plasmids used in this study

| Bacterial strain or plasmids | Principal characteristics | Source or References |
| --- | --- | --- |
| **Strain** |  |  |
| *E. coli* DH5α | SupE44ΔlacU169(φ80 lacZΔM15) hsdR17 recA1 endA1 gyrA96 thi-1 relA1, plasmid-free. | TaKaRa |
| *E. coli* BL21(DE3) | F-ompT hsdSB (rB- mB-) gal dcm (DE3), plasmid-free. | Shanghai Weidi |
| *L. lactis* NZ9000 | Derivate strain of MG1363, with nisR and nisK genes for nisin induction, plasmid-free strain. | NIZO |
| *E. faecalis* MDXEF-1 | Enterococcus faecalis isolated from chicken， microbial preservation number: CCTCC No: M 201488. | Preserved by our laboratory |
| *E. coli* DH5α/pMD19T-Fiber2 | With plasmid pMD19T-Fiber2 in *E. coli* DH5α. | This study |
| *E. coli* BL21(DE3)/pET30a-Fiber2 | With plasmid pET30a-Fiber2 in *E. coli* BL21(DE3). | This study |
| *L. lactis*/pTX8048 | With plasmid pTX8048 in *L. lactis* cremoris NZ9000. | This study |
| *E. faecalis*/pTX8048 | With plasmid pTX8048 in *E. faecalis* MDXEF-1. | This study |
| *L. lactis*/pTX8048- Fiber2-CWA | With plasmid pTX8048-Fiber2-CWA in *L. lactis* cremoris NZ9000. | This study |
| *L. lactis*/pTX8048-DCpep-Fiber2-CWA | With plasmid pTX8048-DC-Fiber2-CWA in *L. lactis* cremoris NZ9000. | This study |
| *E. faecalis*/pTX8048- Fiber2-CWA | With plasmid pTX8048-Fiber2-CWA in *E. faecalis* MDXEF-1. | This study |
| *E. faecalis*/pTX8048-DCpep-Fiber2-CWA | With plasmid pTX8048-DC-Fiber2-CWA in *E. faecalis* MDXEF-1. | This study |
| **Plasmid** |  |  |
| pMD19T | High-efficiency TA cloning vector. | TaKaRa |
| pET30a | Escherichia coli expression vector. | Solarbio |
| pMD19T-Fiber2 | With fragment encoding Fiber2 protein in pMD19T. | This study |
| pET30a-Fiber2 | With fragment encoding Fiber2 protein in pET30a. | This study |
| pTX8048 | With fragment encoding signal peptide of secretion protein Usp45 (SP). | (39) |
| pTX8048-Fiber2-CWA | With fragment encoding signal peptide of secretion protein Usp45 (SP) and Fiber2 protein in anchored form, no dendritic cell targeting peptides. | This study |
| pTX8048-DC-Fiber2-CWA | With fragment encoding signal peptide of secretion protein Usp45 (SP) and Fiber2 protein in anchored form, contains dendritic cell targeting peptides. | This study |

Table S2 Primer sequences with their corresponding PCR product size.

| Name of primer | GenBank Accession | Primer sequences (5' to 3') | | Enzyme | Product size | Source or References |
| --- | --- | --- | --- | --- | --- | --- |
| Fiber2-F1(pET) | / | | CGCGGATCC ATGGCGGCCCTCACG | *Bam*H I | 1440 bp | This study |
| Fiber2-R1(pET) | / | | CCC CTCGAG TTACACGGCGTTGCCTG | *Xho* I | 1440 bp | This study |
| Fiber2-F2(pTX) | / | | CGCGGATCCATGTTAAGAGCTCCTAAAAGACG | *Bam*H I | 1440 bp | This study |
| Fiber2-R2(pTX) | / | | GGGGTACCTGGTAAACTAGCAGCAGGACAAG | *Kpn*I | 1440 bp | This study |
| 52K-F | HE603114.1 | | ATGGCGCAGATGGCTAAGG | / | 176 bp | (41) |
| 52K-R | HE603114.1 | | AGCGCCTGGGTCAAACCGA | / | 176 bp | (41) |
| IL-2-F | HQ739081.1 | | GTGGCTAACTAATCTGCTGTCC | / | 105 bp | (23) |
| IL-2-R | HQ739081.1 | | GTAGGGCTTACAGAAAGGATCAA | / | 105 bp | (23) |
| IL-4-F | GU119892.1 | | CTGTGCCCACGCTGTGCTTA | / | 83 bp | This study |
| IL-4-R | GU119892.1 | | GGAAACCTCTCCCTGGATGTCA | / | 83 bp | This study |
| IL-6-F | HM179640.1 | | CCAGAAATCCCTCCTCGCCAATC |  | 111 bp | (63) |
| IL-6-R | HM179640.1 | | GCCCTCACGGTCTTCTCCATAAAC |  | 111 bp | (63) |
| IL-10-F | AJ621614.1 | | GGCTCACTTCCTCCTCC | / | 112 bp | This study |
| IL-10-R | AJ621614.1 | | TGACTTTCACCTGCAGATG | / | 112 bp | This study |
| IL-17A-F | HQ008777.1 | | CCATTCCAGGTGCGTGAACT |  | 130 bp | This study |
| IL-17A-R | HQ008777.1 | | TTTCTTCTCCAGGCGGTACG |  | 130 bp | This study |
| IFN-γ-F | FJ538013.1 | | CAAAGCCGCACATCAAACA | / | 80 bp | (23) |
| IFN-γ-R | FJ538013.1 | | TTTCACCTTCTTCACGCCATC | / | 80 bp | (23) |
| β-actin-F | NM_205518.2 | | GCCAACAGAGAGAAGATGACAC | / | 138 bp | (26) |
| β-actin-R | NM_205518.2 | | GTAACACCATCACCAGAGTCCA | / | 138 bp | (26) |

# Supplementary Figures

Figure S1 Western blot detection of viral proteins


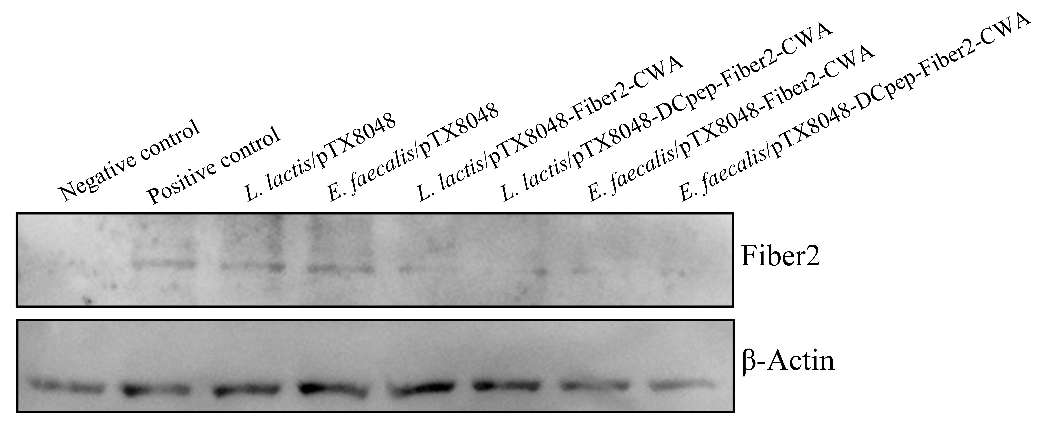


Figure S2 Gross examination of heart, spleen, and kidney from chickens.


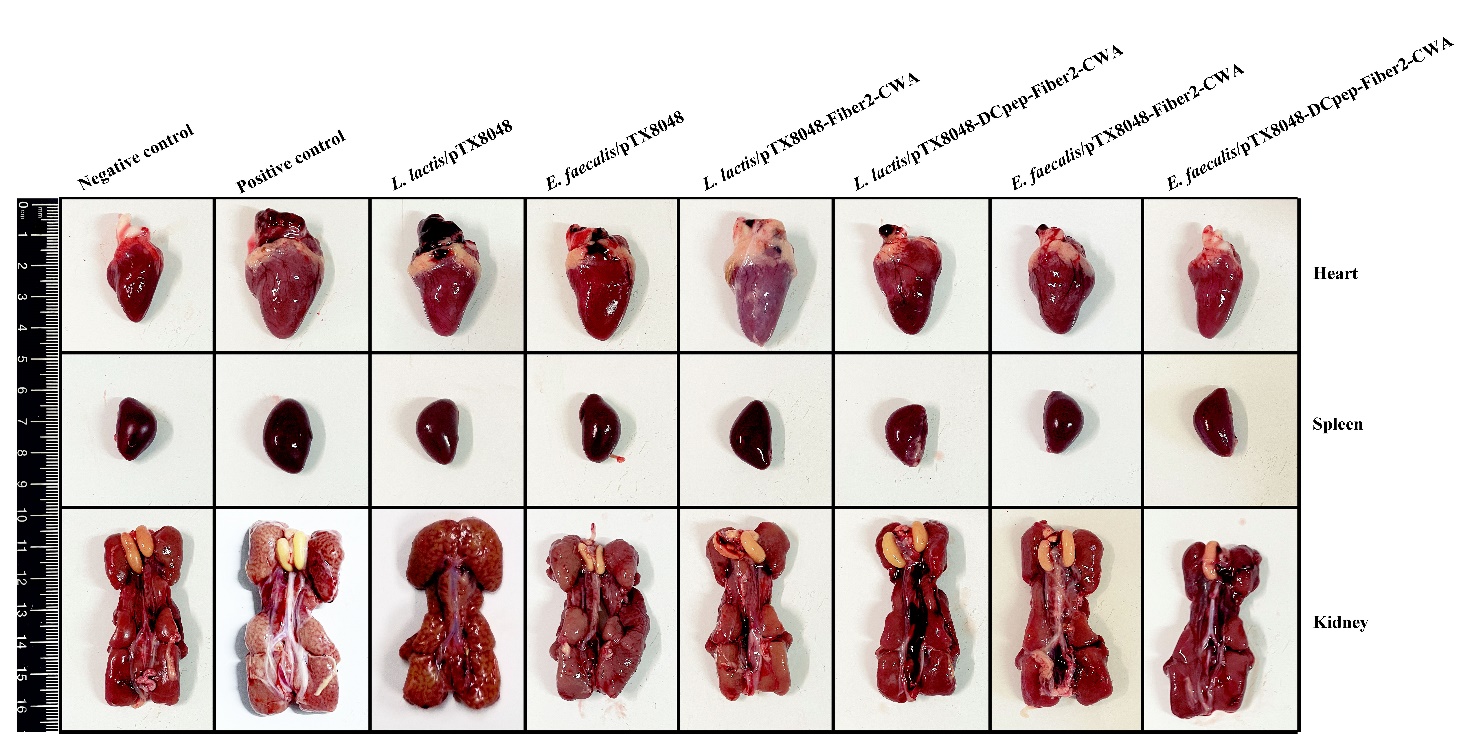

Supplement: Supplementary Figure 1 — Western blot detection of viral proteins. Detection of viral protein in liver tissues from each group on the 5dpi. Fiber2 polyclonal antibody (1:2000) and HRP-labeled goat anti-rabbit secondary antibody (1:2000) were used for detection. β-actin was used as an internal reference for detection. The amount of viral protein in the four recombinant lactic acid bacteria immunization groups was lower than that in the control group. [file DataSheet_1.docx]
